# Smoking control in Brazil: a public health success story

**DOI:** 10.1590/1516-3180.2017.1353230417

**Published:** 2017-04-03

**Authors:** Paulo Andrade Lotufo

**Affiliations:** I MD, DrPH. Full Professor, Department of Internal Medicine, Faculdade de Medicina da Universidade de São Paulo (FMUSP), São Paulo (SP), Brazil.

The Brazilian national football team’s defeat by Germany during the 2014 World Cup, by a vexatious score of 7-1, was not the worst misery for Brazilians over the last three years. During this same period, an organized plot among federal officials, politicians and chief executive officers (CEOs) of major companies, involving a complex exchange of overpriced government contracts relating to engineering and construction, engineering and energy, with bribery and illegal money for electoral campaigns, was revealed.

The national TV networks have broadcast every detail of the investigation reports from both the federal police and the Brazilian Attorney-General’s office, and also from the court trials. The fallout so far is that the President of the Republic (in her second term) was impeached, and the House Speaker and also the former Governor of Rio de Janeiro, who are also two of the wealthiest men in the country, are in jail. All of this is combined with the worst economic recession since the early 1900s. Moreover, in the field of public health, there is a feeling of going back to the past. The Zika epidemic emerged in Brazil, deaths due to pertussis have risen and yellow fever is marching toward urban settings. Consequently, a feeling of discouragement, bewilderment, anger and disenchantment has beset Brazilians and has blurred the vision of any possible improvement in society.

**Figure 1:**
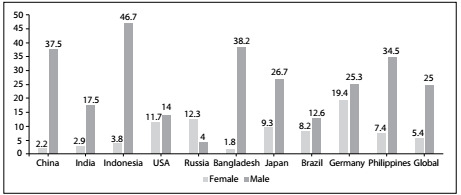
Age-adjusted prevalence rates (in %) for smoking habit among the 10 countries with the highest numbers of smokers, according to the Global Burden of Disease study.^1^

**Figure 2:**
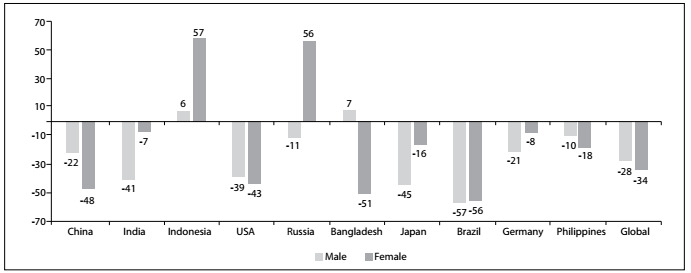
Difference (%) in age-adjusted prevalence rates for smoking habit between 1990 and 2015 among the 10 countries with the highest numbers of smokers, according to the Global Burden of Disease study.^1^

Nonetheless, one piece of good news in the field of chronic diseases stands out: faster decline in smoking prevalence among the most populous countries in the world. The Global Burden of Diseases 2015 Tobacco Collaborators published an extensive paper addressing the prevalence of smoking and the attributable disease burden in 195 countries from 1990 to 2015. From this major paper published in the Lancet,^1^ we can focus on comparison of the 10 countries with most people smoking in 1990, i.e. China, India, Indonesia, Russia, United States, Bangladesh, Japan, Brazil, Germany and the Philippines. **Figure 1** shows that among these countries, in 2015, Brazil had the lowest prevalence for both sexes and the lowest for men. The trends over these 25 years were very remarkable in most of the top 10 countries, but the drop in the prevalence rate was most impressive in Brazil with a 55% reduction, as shown in **Figure 2**.

Despite the horrible political situation, some Brazilians believe that this process looks like “creative destruction” of the democratic order, which may lead to reinvigorated institutions in the future. Whether optimistic or not about politics, most Brazilians should be proud of this reduction in the burden of chronic diseases due to diminution of the risk factor of the smoking habit.
